# Emotional loneliness, perceived stress, and academic burnout of medical students after the COVID-19 pandemic

**DOI:** 10.3389/fpsyg.2024.1370845

**Published:** 2024-07-23

**Authors:** Cem Malakcioglu

**Affiliations:** Medical School, Department of Medical Education, İstanbul Medeniyet University, Istanbul, Türkiye

**Keywords:** loneliness, stress, burnout, psychological resilience, medical students

## Abstract

**Introduction:**

In recent times, emotional loneliness has been increasing among young people, despite their intense social interaction via virtual platforms and in real life. Their social-emotional development seems to be interrupted due to high levels of psychological stress, and it probably results in significant academic problems. This study aimed to investigate the relationships between loneliness, perceived stress, and academic burnout among medical students after the COVID-19 pandemic.

**Methods:**

Datasets were collected from 630 medical students (51.7% women, with the mean age of 21.31 and the standard deviation of 2.14) by using scales for emotional and social loneliness, psychological stress, and academic burnout in January 2023. After verifying normality, a *t*-test and ANOVA were used to compare groups. Pearson's correlation coefficient and path analyses were also utilized for data analysis.

**Results:**

In terms of loneliness, there were significant differences between genders in preclinical and clinical term groups, and stress levels were also significantly different between the two term groups. Men were found to be more emotionally lonely than women. No significant difference was observed for academic burnout across gender and term groups. Perceived stress played a mediator role between emotional loneliness and academic burnout, while social loneliness had almost no effect on either stress or academic burnout. Stress increased significantly as educational levels increased.

**Conclusions:**

According to the results, emotional loneliness and stress increase academic burnout. To alleviate burnout, emotional loneliness and stress should be decreased through various psychosocial interventions, such as group therapies. Addressing the psychological issues and improving the psychological resilience of medical students can also help.

## Introduction

During the COVID-19 pandemic, schools and universities were shut down around the world for nearly 1.5 years. At the university level, students continued to take online courses as an alternative to or in addition to face-to-face education. Distance education may have provided students with much theoretical knowledge; however, the social and emotional needs of students were mostly not met during lockdowns. Research showed that students experienced loneliness despite being able to connect with others and attend online courses through technology (Werner et al., 2021). Increased technology use (e.g., Internet and smart-phone use) contributes to loneliness by distancing people from real-life emotional interactions (Richardson et al., 2017). Moreover, loneliness is connected to several other negative emotions and mental health issues during pandemics and other crises, such as natural disasters and war (Bao et al., 2021). Depression and other affective disorders are highly associated with loneliness (Whiston et al., 2022), as well as sadness and worry.

### Loneliness

There are different types and dimensions; the best and most widely known ones are emotional and social loneliness. Loneliness is both an emotion and a social situation. However, emotional rather than social loneliness was more prevalent among young people during COVID-19 because the only way they were able to connect with others was via social media and other online platforms, such as online games (Houghton et al., 2022). Although they could socially interact with others around the world virtually, they still experienced loneliness to a great extent (Hammoud et al., 2021). Physical separation does not necessarily lead to social isolation from others, but emotional loneliness can occur despite physical or social closeness.

### Effects of loneliness

There are some serious effects of loneliness. Prolonged loneliness can lead to the development of social anxiety and phobias (Chen et al., 2021). Moreover, prolonged loneliness can cause some neurological damage, which can lead to serious neuropsychiatric problems. For example, a disruption in the processing of emotional faces causes people to become more responsive to sadness and anxiety (Cheeta et al., 2021). Loneliness can also cause addiction issues (Ingram et al., 2020). Substances are commonly used to ineffectively deal with psychological problems. Many studies have observed a relationship between stress and loneliness, especially the predictive role of loneliness on stress (Landmann and Rohmann, 2022). Social distancing measures caused inner and interpersonal distress, that is, talking on the phone and chatting in front of the camera are not the same as face-to-face real emotional interactions. Deep emotional needs are largely met through real-life experiences perceived by all senses.

Intensive and intimate peer interaction is essential for the social and emotional development of children, adolescents, and even young adults (Hoffart et al., 2022). Young people learn to cope with stress and other psychological difficulties by observing each other naturally and without any restrictions such as wearing a mask or turning off the microphone or camera, which prevent verbal and/or non-verbal communication. For university students, it is also important to form strong emotional bonds and intimate relationships with their peers and older adults (Holmstrom et al., 2022). Distance education does not adequately improve the psychological resilience of students, such as coping with stress (Hoffart et al., 2022). For example, real-life social interactions with teachers and older students can serve as functional role models for students to cope with stress more effectively, especially if they are from dysfunctional families or other adverse social environments.

### Pandemic and consequences

There are several recent studies about the detrimental consequences of emotional loneliness and stress in university students during and after the COVID-19 pandemic. Medical students, in particular, have been affected by these issues, perhaps more so than others due to their professional interests. Consequently, many of them are questioning their career plans due to numerous stress factors related to pandemic conditions (Zheng et al., 2021). One of the main concerns is the changing professional values and perfectionist academic self-expectations, as well as high expectations from others. Academic stress has been very high among medical students, and the stress is usually accompanied by low academic satisfaction (Bulut et al., 2021). Therefore, medical students appear to be at risk of academic burnout more than ever due to the COVID-19 pandemic and its aftermath. Academic burnout among medical students has been commonly observed by faculty members in medical schools across different institutions, cities, and countries. One significant risk factor may be loneliness, especially emotional loneliness.

This study aimed to investigate the relationship between loneliness, perceived stress, and academic burnout in medical students during the COVID-19 pandemic. The specific objectives were as follows: (1) correlations between research variables, (2) differences according to gender and terms (preclinical and clinical groups), (3) effects of loneliness and perceived stress on academic burnout, and (4) changes across groups of six terms in the medical school.

## Method

### Participants

This study was conducted in the Medical School at İstanbul Medeniyet University. A total of 667 students (51.7% women, with a mean age of 21.31 and standard deviation of 2.14) volunteered and filled out the online forms completely and appropriately, comprising almost two-thirds of the student body (*n* = 1,071) in the medical school. However, 37 forms that were inappropriate or incomplete were discarded and excluded from the data analysis. Therefore, the completion rate of the online form was 94.4%. The sample size was calculated as 283 with a 95% confidence interval. Evidently, the participation was much better than expected, and the results are generalizable to similar populations. Several announcements were made to all students, and their voluntary participation was encouraged. All participants were at least 18 years old and above (ranging between 18 and 29 years). Participation rates were also balanced according to gender; a close number of men and women participated in this study.

### Data collection and instruments

Online forms were used to collect data due to low face-to-face class attendance numbers after the COVID-19 pandemic. The form included informed consent and brief demographic information sections, as well as scales and questions related to loneliness, stress, and academic burnout. A Turkish adaptation of the De Jong Gierveld Loneliness Scale (Gierveld and Tilburg, 2006; Cavdar et al., 2015) was used to measure the loneliness (L) levels of the participants. This scale includes six items in total: the emotional loneliness (EL) scale with three items and the social loneliness (SL) scale with three items. A three-point rating scale is used to state the frequency of loneliness: 0 indicates “No,” 1 indicates “more or less,” and 2 indicates “yes.” Higher scores indicate higher levels of loneliness. In this study, Cronbach's alpha values, which were considered the internal consistency reliability coefficient, were 0.81 for the total L scores (six items), 0.75 for the EL scores, and 0.76 for the SL scores. A Turkish adaptation of the Perceived Stress Scale with 10 items (Cohen et al., 1983; Eskin et al., 2013) was used to measure perceived stress within the last month (PS) in two dimensions: the Perceived distress (PD) scale with six items and the perceived low self-efficacy (PLSE) scale with four items. A five-point Likert-type rating scale was used, with 0 indicating “never,” 1 indicating “almost never,” 2 indicating “sometimes,” 3 indicating “often,” and 4 indicating “very often.” High scores indicated high stress. Cronbach's alpha values for the current study were 0.90 for the total PS scores (10 items), 0.82 for the PD scores (six items), and 0.80 for the PLSE scores (four items). A Turkish adaptation of the Maslach Burnout Inventory-Student Scale (MBI-SS, 13 items; Schaufeli et al., 2002; Capri et al., 2011) was used to measure academic burnout (AB) in three factors: the exhaustion (E) scale with five items, the cynicism (C) scale with four items, and the low academic efficacy (LAE) scale with four items. A six-point Likert-type scale was used from 0, indicating “never,” to 5, indicating “always.” Higher scores indicated a higher level of burnout. Cronbach's alpha values were 0.92 for the total AB scores (13 items), 0.86 for the E scores (five items), 0.81 for the C scores (four items), and 0.77 for the LAE scores (four items).

### Procedure

This study analyzed the relationship between loneliness types, perceived stress, and academic burnout in medical students. Data were collected in January 2023. The İstanbul Medeniyet University Clinical Research Ethics Committee provided approval (Date: 06.10.2021, Decision number: 2021/0501) for this study; institutional permits were taken from the dean's office in advance. Written informed consent was obtained from all participants following the approval from the committee. All procedures were carried out in accordance with the Declaration of Helsinki.

### Data analysis

The Kolmogorov–Smirnov test was conducted to test the normality of variables. No preliminary analysis was required since the variable distributions were not statistically significant compared to the normal distributions. All tests were two-tailed. Pearson's correlation coefficient was used for relationship testing, and an independent samples *t*-test was conducted for binary group comparisons. For multiple comparisons between the six terms, a one-way ANOVA with a Tukey adaptation of a *post-hoc* test was conducted. The analysis of residuals confirmed the assumptions of linearity. The homogeneity of variances was verified by conducting Levene's test. There were no very high correlations between the variables; all of them were below 0.8. After the verification of other required assumptions (e.g., multivariate linearity through scatter plot matrices and Q-Q plots), path analyses were conducted to investigate the relationships between model variables. The sample size adequacy was tested by conducting the Kaiser–Meyer–Olkin test (KMO = 0.896). The minimum statistical significance was determined at the level of 0.05. For regression models, standardized weights were reported. Statistical analyses were performed using SPSS 25 and AMOS 24 software programs (IBM, USA).

## Results

### Correlations between research variables

There were no missing data or any outliers. Descriptive information about the categorical and continuous variables in this study is shown in Table 1. Since the values were distributed almost normally, Pearson's correlation coefficients were calculated and are shown in Table 2.

**Table 1:** Descriptive information.

**Categorical variables**	***N***	**%**	**Continuous variables**	**M**	**SD**
Gender			Age	21.31	2.14
Women	326	51.7	Model variables		
Men	304	48.3	Emotional loneliness (EL)	3.49	1.12
Terms			Social loneliness (SL)	3.46	1.13
Term 1	122	19.4	Loneliness (L)	6.96	1.76
Term 2	113	17.9	Perceived distress (PD)	13.64	3.53
Term 3	120	19.1	Perceived low self-efficacy (PLSE)	10.62	3.70
Term 4	90	14.2	Perceived stress (PS)	24.26	8.37
Term 5	93	14.8	Exhaustion (E)	11.86	4.85
Term 6	92	14.6	Cynicism (C)	10.22	3.51
Preclinical (terms 1–3)	355	56.4	Low academic efficacy (LAE)	10.24	3.16
Clinical (terms 4–6)	275	43.6	Academic burnout (AB)	32.32	9.40

M, Mean; SD, Standard deviation.

**Table 2:** Correlations (Pearson's correlation coefficients) between research variables.

	**Loneliness**			**Stress**			**Burnout**			
	**EL**	**SL**	**L**	**PD**	**PLSE**	**PS**	**E**	**C**	**LAE**	**AB**
Age	−0.133^*^	0.007	−0.082^*^	0.419^**^	0.386^**^	0.465^**^	−0.021	0.025	0.013	0.003
EL		0.059	0.722^**^	0.312^**^	0.317^**^	0.358^**^	−0.015	0.167^*^	0.154^*^	0.258^**^
SL			0.624^**^	0.084^*^	0.088^*^	0.089^*^	−0.057	−0.037	−0.014	−0.026
L				0.219^**^	0.218^**^	0.250^**^	−0.044	0.152^*^	0.107^*^	0.155^*^
PD					0.497^**^	0.733^**^	0.609^**^	0.554^**^	0.133^*^	0.536^**^
PLSE						0.675^**^	0.420^**^	0.430^**^	0.127^*^	0.412^**^
PS							0.514^**^	0.502^**^	0.113^*^	0.631^**^
E								0.519^**^	0.456^**^	0.767^**^
C									0.472^**^	0.699^**^
LAE										0.754^**^

^*^p < 0.05.

^**^p < 0.01.

EL, Emotional Loneliness; SL, Social Loneliness; L, Loneliness; PD, Perceived Distress; PLSE, Perceived Low Self-Efficacy; PS, Perceived Stress; E, Exhaustion; C, Cynicism; LAE, Low Academic Efficacy; AB, Academic Burnout.

### Differences according to gender and terms (preclinical and clinical groups)

Loneliness, stress, and academic burnout variables were compared according to gender and terms (Table 3). When two groups, namely the preclinical (terms 1, 2, and 3) and clinical (terms 4, 5, and 6), were created, significant differences were observed between these groups regarding EL (*p* = 0.045), SL (*p* = 0.041), PD (*p* = 0.000), PLSE (*p* = 0.000), and PS (*p* = 0.000) according to the independent samples *t*-test. Among them, the preclinical group had significantly higher scores only for SL. For the gender groups, only the scores for EL (*p* = 0.006) and L (*p* = 0.018) were significantly different as men had higher scores in both. Other comparisons did not yield any significant difference between the groups (Table 3). The effect sizes of comparisons (Cohen's *d* coefficients) ranged from 0.506 to 0.849, indicating medium to large effects.

**Table 3:** Differences according to gender and terms (preclinical and clinical) groups.

			**Gender**					**Term**				
			**Women**		**Men**			**Preclinical**		**Clinical**	
	**Min**.	**Max**.	**M**	**SD**	**M**	**SD**	***p***	**M**	**SD**	**M**	**SD**	***p***
EL	0	6	3.26	0.95	3.73	1.26	0.006	3.19	0.95	3.63	1.23	0.045
SL	0	6	3.34	0.91	3.58	1.15	0.301	3.74	0.99	3.24	0.81	0.041
L	0	12	6.60	1.68	7.31	1.83	0.018	6.93	1.75	6.87	1.72	0.571
PD	0	24	14.11	3.83	13.17	3.23	0.072	11.60	3.92	16.31	3.48	0.000
PLSE	0	16	10.53	3.75	10.70	3.64	0.557	9.61	3.56	11.91	3.47	0.000
PS	0	40	24.64	8.38	23.87	8.35	0.284	21.21	8.48	28.22	7.97	0.000
E	0	25	12.12	4.99	11.60	4.70	0.173	11.98	4.97	12.15	4.71	0.546
C	0	20	10.25	3.37	10.18	3.64	0.801	10.29	3.60	10.23	3.57	0.813
LAE	0	20	10.45	3.18	10.03	3.17	0.096	10.40	3.25	10.06	3.08	0.181
AB	0	65	32.82	9.54	31.81	9.25	0.174	32.67	9.73	32.44	9.68	0.495

M, Mean; SD, Standard Deviation; EL, Emotional Loneliness; SL, Social Loneliness; L, Loneliness; PD, Perceived Distress; PLSE, Perceived Low Self-Efficacy; PS, Perceived Stress; E, Exhaustion; C, Cynicism; LAE, Low Academic Efficacy; AB, Academic Burnout.

### Effects of loneliness and perceived stress on academic burnout

**Figure 1:**
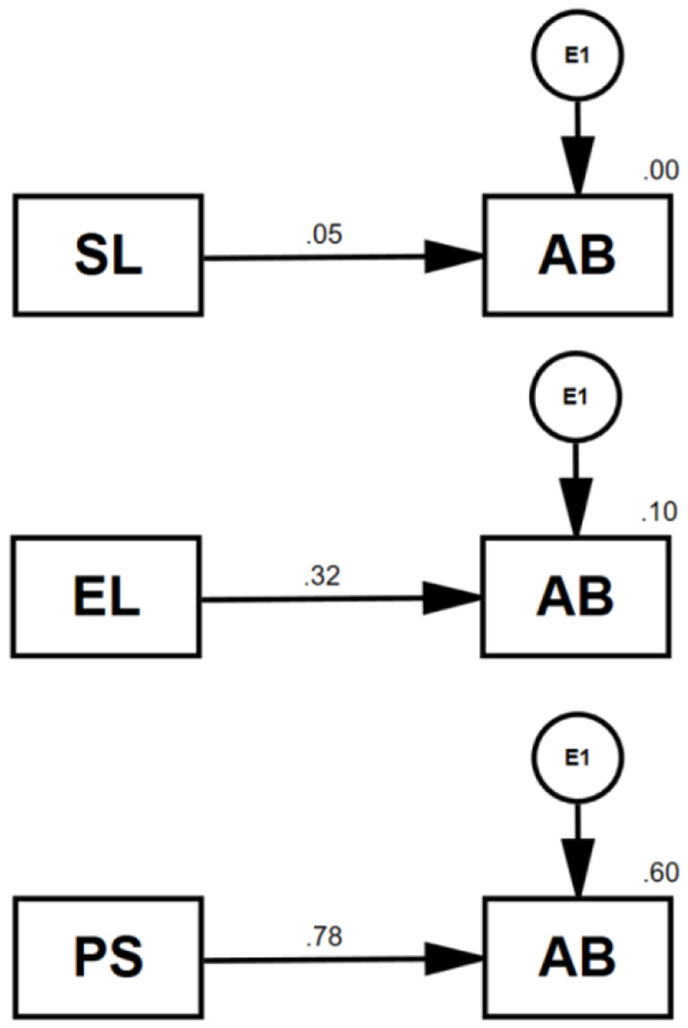
Path analyses between loneliness dimensions (SL and EL), perceived stress (PS), and academic burnout (AB).

**Figure 2:**
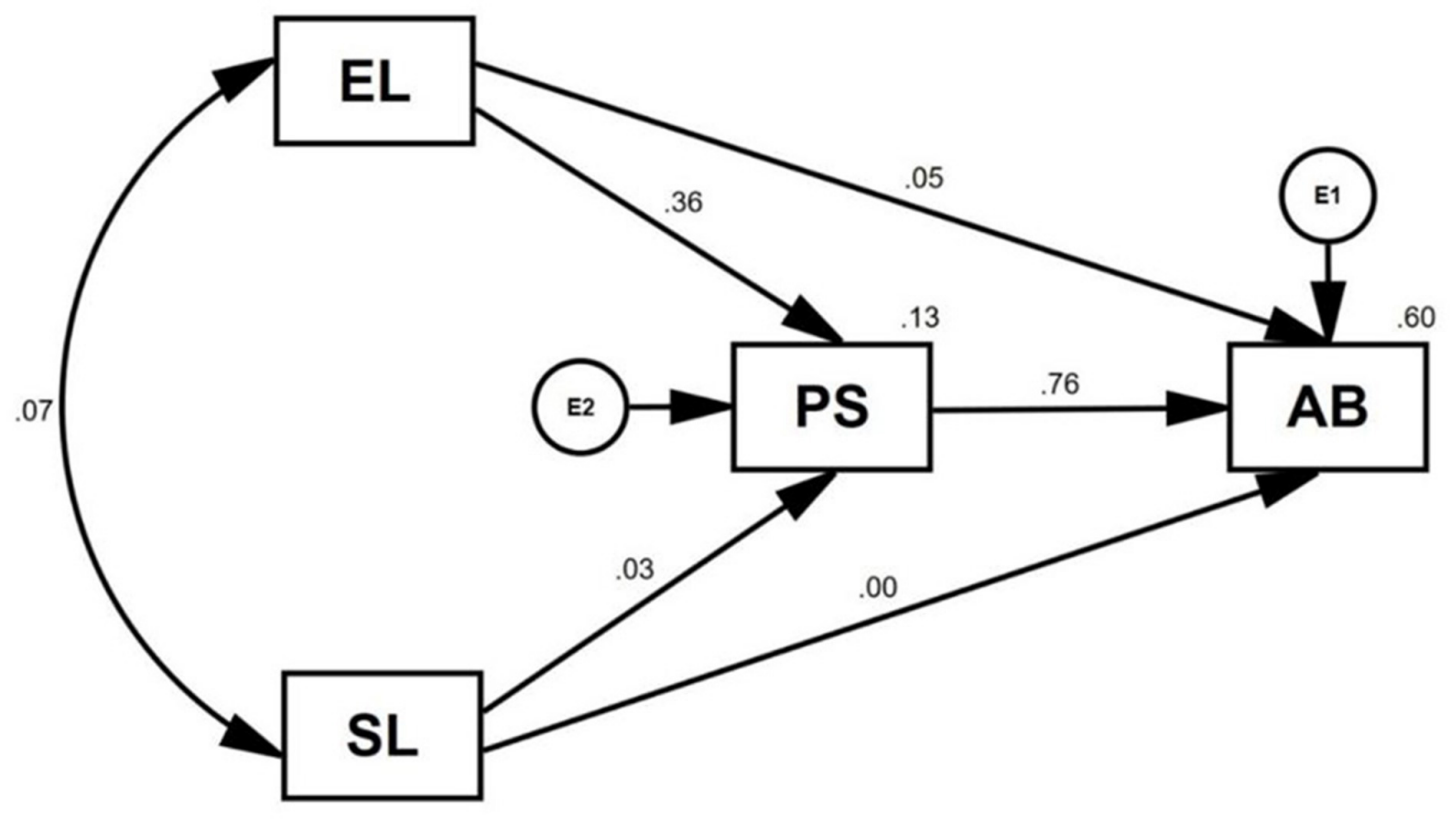
Path analyses for the mediating role of perceived stress (PS) between loneliness dimensions (SL and EL) and academic burnout (AB).

The standardized effects of PS (β = 0.783, *p* < 0.01), EL (β = 0.324, *p* < 0.01), and SL (β = 0.052) on AB were examined by separate path (linear regression) analyses (Figure 1). While the increase in EL resulted in a 10% increase in AB (*R*^2^ = 0.10), SL had almost no effect. On the contrary, the single effect of PS on AB was 60% (*R*^2^ = 0.60).A mediation model was built to examine their combined effects on AB through the mediating role of PS (Figure 2).

In this mediation model, the effect of SL on PS was minute (β = 0.031) and insignificant, while the effect of EL on PS was significant (β = 0.364, *p* < 0.01). However, the effect of EL on AB became insignificant (β = 0.054) with the addition of PS in the model. Thus, EL affected AB indirectly through PS to a large extent. Therefore, PS mediated the relationship between EL and AB. In other words, EL increased PS and, accordingly, PS increased AB. The acceptable results of the data-fit indices for this structural equation model are shown in Table 4.

**Table 4:** Goodness-of-fit statistics for the mediation model.

	**χ^2^/df**	**RMSEA**	**SRMR**	**TLI**	**CFI**
Model for the mediating role of perceived stress between dimensions of loneliness and academic burnout	2.34	0.079	0.074	0.912	0.919
Good fit	<2	<0.05	<0.05	>0.95	>0.95
Acceptable fit	<3	<0.1	<0.08	>0.90	>0.90

RMSEA, Root Mean Square Error of Approximation; SRMR, Standardized Root Mean Squared Residual; TLI, Tucker-Lewis Index; CFI, Comparative Fit Index.

### Changes across groups of six terms in the medical school

**Figure 3:**
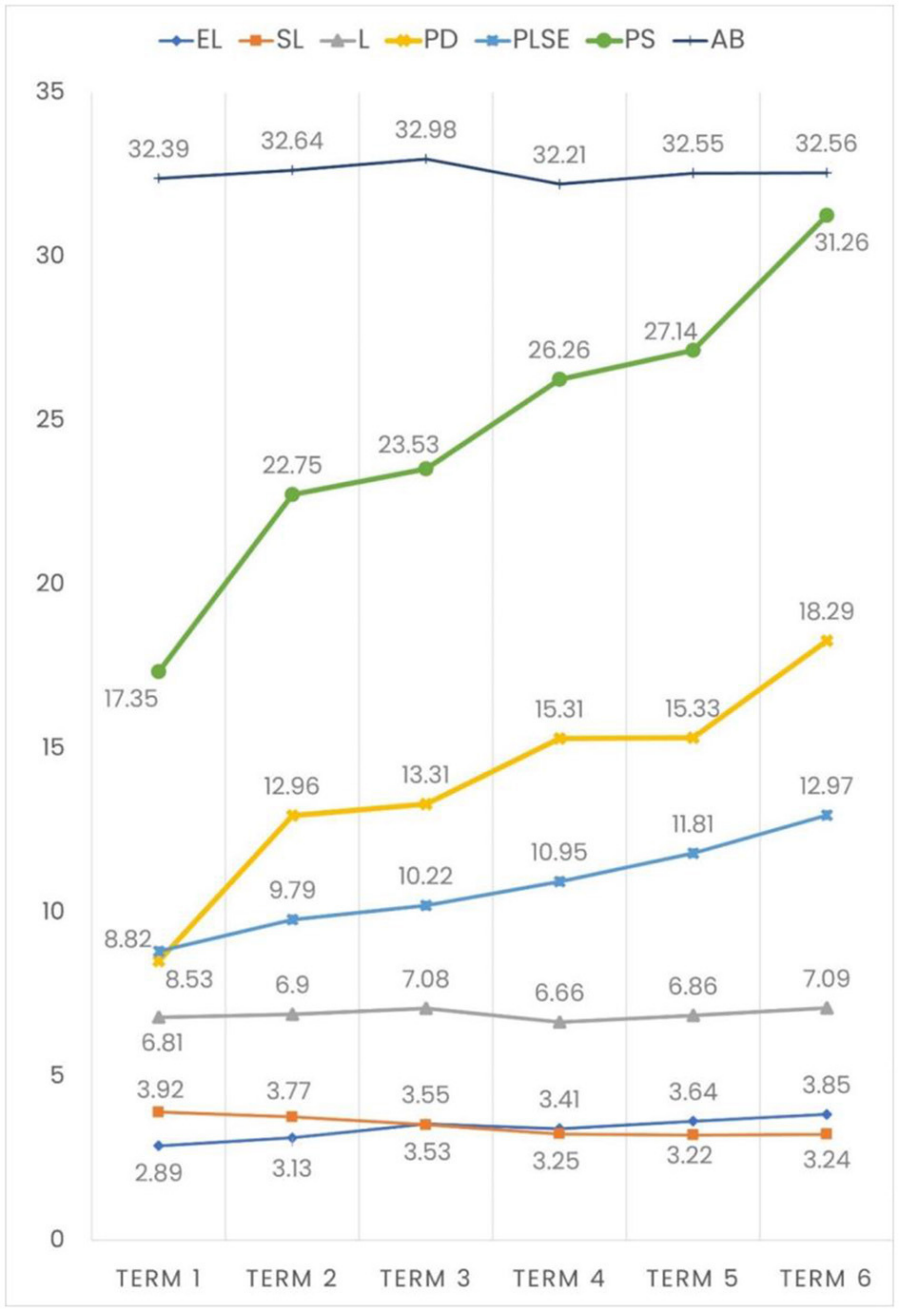
Change in the trends of the AB, L, and PS variables' means according to the terms (EL, Emotional Loneliness; SL, Social Loneliness; L, Loneliness; PD, Perceived Distress; PLSE, Perceived Low Self-Efficacy; PS, Perceived Stress; AB, Academic Burnout).

As shown in Figure 3, AB and L scores were observed to be equal across the terms, despite their slight fluctuations. However, PS with both its dimensions (PD and PLSE) significantly increased across terms, and the mean of PS almost doubled from 17.35 to 31.26 (Figure 3). This was one of the major findings of the current study. The ANOVA results showed that PS and its two dimensions (PD and PLSE) significantly increased across terms (Table 5). Similarly, EL also increased along with an increase in terms. Multiple comparisons with the Tukey adaptation of a post-hoc test verified these findings across terms (*R*^2^ = 0.10). There was a final question at the end to decide and plan for the intervention: “Would you like to participate in stress management workshops in the coming months?” A total of 212 students (33.7%) responded with “Yes.”

**Table 5:** One-way ANOVA results across the groups of six terms.

	**SS_w_**	**SS_b_**	**MS_w_**	**MS_b_**	**F**	***p***
EL	2,657.583	1,630.789	4.259	32.758	7.692	0.000
SL	5,321.039	670.622	8.527	13.524	1.586	0.162
L	8,462.764	283.610	13.562	56.722	2.182	0.071
PD	20,296.420	6,401.207	32.526	1,280.24	39.360	0.000
PLSE	7,306.892	1,300.379	11.710	260.076	22.210	0.000
PS	37,523.689	12,859.353	60.134	2,571.871	42.769	0.000
E	14,560.974	292.120	23.335	58.424	2.504	0.059
C	7,605.996	108.336	12.189	21.667	1.778	0.115
LAE	6,315.734	280.641	10.121	5.728	0.566	0.726
AB	55,027.927	636.638	88.186	127.328	1.444	0.207

W, within-group variances; B, between-groups variances; df_b_ = 5, df_w_ = 624. Goodness of fit (between groups/total variance) was confirmed for significant differences (R^2^ > 0.24). Tukey's *post-hoc* test was conducted for multiple comparisons, and significant increases between groups were observed as the terms increased.

## Discussion

### Correlations between research variables

Many recent studies have observed a high prevalence of and a remarkable increase in emotional loneliness (Dahlberg, 2021), stress (White, 2022), and academic burnout (Forycka et al., 2022) among young people during COVID-19. These variables were almost normally distributed in the present study, and there were very strong significant correlations between them in accordance with recent literature. Loneliness is a negative emotional state related to many mental health problems, and young people such as university students have been at risk of emotional loneliness during and after COVID-19, as evident from the findings of this study. Despite not being socially separated from others as they could communicate through technology, connect to others via social media, spend time with their relatives, among other things, they felt lonely. Although people were not socially isolated from others, they became emotionally more lonely, and their stress levels increased partly due to negative feelings such as loneliness.

### Differences according to gender and terms (preclinical and clinical groups)

Recent studies have observed that men generally feel more lonely than women all around the world; younger men are the highest risk group (Barretoa et al., 2021). This finding was also supported by the current study (Table 3). Men were generally observed to be more hesitant to express their problems and seek help, and the pandemic conditions most likely exacerbated the situation. For example, it was locally observed that academic success rates were lower among male students than among female students. Contrarily, it was also observed that female students were asking for help more than male students from their medical teachers. Moreover, COVID-19 seemed to have caused other serious academic problems among university students, such as low self-confidence (Malakcioglu, 2020a), perfectionism (Malakcioglu, 2020b), and procrastination (Malakcioglu, 2020c), in addition to social and emotional problems such as loneliness and psychological stress.

Another significant finding was the difference in the types of loneliness between the preclinical and clinical groups. While emotional loneliness was higher in students from the clinical group, students from the preclinical group experienced more social loneliness. During the COVID-19 pandemic, some students from the clinical group had to spend time in hospitals for practicums, whereas preclinical students could not attend most of the courses with their peers. The increased level of emotional loneliness and the decreased level of social loneliness can be attributed to the higher levels of perceived stress in clinical students. Indeed, hospitals are stressful environments, especially during pandemics. Healthcare professionals experience excessive stress and become burned out while their psychological resilience diminishes. In particular, those working in emergency departments and those with less experience are more negatively influenced (Corlade-Andrei et al., 2022). Medical students who have been practicing in hospitals are among the least experienced and the most burned out (Kannampallil et al., 2020).

### Effects of loneliness and perceived stress on academic burnout

Burnout and stress can emerge from and be fed by many sources, but intimate emotional bonds help one overcome stressful life events such as pandemics. This study showed that both emotional loneliness and perceived stress have significant direct and indirect effects on academic burnout in medical students (Figures 1, 2). Medical students affected by pandemics require not only physiological treatments but also psychological interventions to increase resilience. Fortunately, mental health providers have started to offer several interventions to decrease negative emotions such as loneliness, worry, and related psychological problems such as depression and anxiety caused by the COVID-19 pandemic (Kanter et al., 2021). Group therapies can be good alternative modalities for strengthening emotional attachments and fighting against burnout risks. Burnout is highly associated with psychological stress (Lou et al., 2022). The results of this study revealed high correlations between loneliness, stress, and burnout. Emotional loneliness, in particular, leads to stress and ultimately causes burnout as it adds up to many other negative factors.

### Changes across groups of six terms in the medical school

The results indicated that academic burnout and loneliness scores did not differ significantly across terms; however, the psychological stress scores of both dimensions (distress and low self-efficacy) did increase significantly (Figure 3). In parallel with the findings of this study, some studies have shown that various difficulties in medical education accumulate and increase the stress factor over time (Richardson et al., 2017). It appears that stress may have an increasing effect on medical students over the years, and new large-scale longitudinal studies are needed to better understand this.

This study aims to inspire researchers to conduct necessary future studies to determine other variables contributing to psychological stress and burnout. Therefore, it is strongly recommended to conduct more research and develop effective interventions to better cope with stress and burnout risk factors caused by the COVID-19 pandemic and other crises. This pandemic is expected to eventually turn into an endemic in all parts of the world, but its complex psychosocial effects will continue to impact humanity. Medical students represent hope for healthcare systems. To foster greater hope, we must dedicate our best efforts to helping medical students and all healthcare workers recover from psychological difficulties.

This study has some limitations and strengths. The specific limitations are as follows. First, it was carried out in a single setting with students from one medical school. Second, data were collected via online forms. Therefore, there is uncertainty about whether the students themselves filled out the forms with complete comprehension and care, despite being informed and warned about these issues in advance. Third, only self-reported data were used; the reliability of measurements depended on the sincerity and honesty of the participants. Finally, research variables were limited. In the future, students from other disciplines should be included, and other relevant variables, such as other stress-related variables, coping mechanisms, and suicidal ideation, should also be examined. This study also has some strengths. For instance, many students were eager to participate in this study. Only ~5% of the forms were incomplete or inappropriate. In addition, scale reliability measures were satisfactory. The relationships between the research variables have been clearly elucidated with focus on potential solutions to the mentioned problems.

According to the findings of this study, psychosocial interventions are necessary. Nearly one-third of the participants marked the “stress management workshop” option as a bonus for this study. Fortunately, the students started receiving benefits from this psychological intervention. There were 10–12 students in each intervention group. In the beginning, a pre-evaluation was performed to identify their stress levels. Active participation of each participant was encouraged. The students expressed themselves, drew pictures of their stress medium, and wrote letters to their stress medium to gain more self-awareness by explaining their thoughts and feelings in more detail. Some relaxation techniques (e.g., mindful breathing) were demonstrated, and they exercised these techniques. Furthermore, they explained how they felt and what they thought about being relaxed. In addition, interactive presentations about psychoeducation were conducted for stress management in each group. The sources and reactions of stress were described and discussed in these sessions. Some recent research findings were shared by the students. At the end of the workshop, their stress levels were measured and evaluated to compare them with their initial stress levels. Some books and movies were also recommended for stress management. It is necessary to adopt a global approach to mental health for this group, considering the statistical figures of the World Health Organization.

## Conclusion

In conclusion, stress management skills are among the professional competencies of physicians. Medical students should be trained in stress management and burnout prevention. The COVID-19 pandemic showed the importance of being psychologically resilient to stressful conditions for everyone, especially for healthcare professionals, many of whom were affected by burnout. Medical students also experienced academic burnout. More research and interventions should be designed and implemented within medical education and other academic disciplines to help students become psychologically resilient together by managing stress skillfully and alleviating burnout by forming stronger emotional bonds.

## Data availability statement

Data is available upon reasonable request from the author.

## Ethics statement

The studies involving humans were approved by İstanbul Medeniyet University Ethics Committee. The studies were conducted in accordance with the local legislation and institutional requirements. The participants provided their written informed consent to participate in this study.

## Author contributions

CM: Writing – original draft, Writing – review & editing.
